# Molecular Organization of the Nanoscale Surface Structures of the Dragonfly *Hemianax papuensis* Wing Epicuticle

**DOI:** 10.1371/journal.pone.0067893

**Published:** 2013-07-09

**Authors:** Elena P. Ivanova, Song Ha Nguyen, Hayden K. Webb, Jafar Hasan, Vi Khanh Truong, Robert N. Lamb, Xiaofei Duan, Mark J. Tobin, Peter J. Mahon, Russell J. Crawford

**Affiliations:** 1 Faculty of Life and Social Sciences, Swinburne University of Technology, Hawthorn, Victoria, Australia; 2 School of Chemistry, The University of Melbourne, Parkville, Victoria, Australia; 3 Australian Synchrotron, Clayton, Victoria, Australia; US Naval Reseach Laboratory, United States of America

## Abstract

The molecular organization of the epicuticle (the outermost layer) of insect wings is vital in the formation of the nanoscale surface patterns that are responsible for bestowing remarkable functional properties. Using a combination of spectroscopic and chromatographic techniques, including Synchrotron-sourced Fourier-transform infrared microspectroscopy (FTIR), x-ray photoelectron spectroscopy (XPS) depth profiling and gas chromatography-mass spectrometry (GCMS), we have identified the chemical components that constitute the nanoscale structures on the surface of the wings of the dragonfly, *Hemianax papuensis*. The major components were identified to be fatty acids, predominantly hexadecanoic acid and octadecanoic acid, and *n*-alkanes with even numbered carbon chains ranging from C_14_ to C_30_. The data obtained from XPS depth profiling, in conjunction with that obtained from GCMS analyses, enabled the location of particular classes of compounds to different regions within the epicuticle. Hexadecanoic acid was found to be a major component of the outer region of the epicuticle, which forms the surface nanostructures, and was also detected in deeper layers along with octadecanoic acid. Aliphatic compounds were detected throughout the epicuticle, and these appeared to form a third discrete layer that was separate from both the inner and outer epicuticles, which has never previously been reported.

## Introduction

Natural surfaces that possess unusual surface properties are increasingly becoming the focus of intensive research [1]–[7]. Superhydrophobic and self-cleaning surfaces are of particular interest in these studies [2], [8], [9]. Recently, it was reported that the wings of several species of insects not only possess superhydrophobic and self-cleaning properties, but also possess strong antibacterial activity [10]. All of these properties are known to arise, at least in part, as a result of the nanoscale structures present on the wing surfaces, however to date the chemical nature and organization of these nanostructures is not fully understood.

The general consensus in the literature is that insect cuticles are composed of a network of chitin and proteins embedded in a matrix of lipids/waxes [11]–[14]. The outermost layer, the epicuticle, is thought to consist only of waxy compounds [11], [15]–[19]. Most previous chemical characterizations of insect epicuticle and/or procuticle (the thicker layer below the epicuticle) physiologies have been performed in (macro) biological contexts [3], [5], [8], [20]–[22]. These studies are typically limited to broad classifications (e.g., waxy compounds) of the insect cuticle and certainly do not examine the specific composition of the epicuticle nanoscale structures [9], [23]–[26]. There is also a lack of knowledge regarding the nature and function of the structural components of the epicuticle and the procuticle.

The epicuticular waxes that form the interfacial layer in contact with the surrounding environment may be considered to be the most relevant structural component of the insect wing [3], [20], [22], [27]–[29]. However, not many studies have been conducted that address the chemistry and topography of the wing together. The most recent work on insect wings to consider both the surface architecture and surface chemistry was conducted by Sun and his colleges [30]. In this study, the chemistry of the wing surface was investigated using XPS to identify fundamental elements present in the elytral surface of beetles. However the molecular identities of the chemical components were not determined.

Hence, the aim of this work was to generate a detailed overview of the compounds that comprise the epicuticle of the wings of the dragonfly *Hemianax papuensis*, together with an understanding of the distribution of compounds throughout the epicuticle. To achieve this aim, a combination of different analytical techniques, i.e., Synchrotron-sourced FTIR, GCMS and XPS depth profiling were employed to investigate the molecular organization of the epicuticular lipids on the wings of the dragonfly *Hemianax papuensis*.

## Materials and Methods

### Materials

*H. papuensis* dragonflies are common inhabitants of the suburban regions of Melbourne, Australia. Specimens were freshly caught and kindly donated by the Melbourne Museum in November 2011. The wings were removed from the body aseptically and stored at room temperature (*ca.* 22°C) in sterile plastic containers until needed.

Chloroform (CHROMASOLV for HPLC) and bis-N,N-(trimethylsilyl) trifluoroacetamide (BSTFA for TMSi derivatives) were obtained from Sigma Aldrich (St. Louis, Missouri, USA), as were the standard alkane solutions (C_8_–C_20_ and C_21_–C_40_) which were employed for GC-MS analyses.

### Extraction of Epicuticular Lipids

Epicuticular lipids were extracted from approximately 0.5 cm^2^ wing sections using chloroform for periods of either 10 seconds or one hour (as optimized in a preliminary study, data not shown). Ten second extractions were used in order to limit the extraction depth to just below the outer epicuticular surface, whereas the extractions over one hour ensured that the entire lipid fraction of the epicuticle was removed from the wing surface. The lipid-chloroform mixture was then filtered through glass wool to remove any particulate contaminants. The lipid fractions were then concentrated by evaporating the solvent, followed by dissolution in half the initial volume of solvent. The chloroform-extracted wings were also retained for further surface characterization and analysis.

### Scanning Electron Microscopy (SEM)

The surface morphologies of the both lipid-extracted and untreated wings were observed using a field emission scanning electron microscope (FeSEM - SUPRA 40VP, Carl Zeiss GmbH, Jena, Germany) at fixed voltage of 3 kV. The wing sample was attached to a metallic substratum using conductive double-sided adhesive tape. Samples were sputter coated with gold using a JEOL NeoCoater (model MP-19020NCTR) prior to imaging, using a method described elsewhere [10]. To observe the structure of cross-sections of the wing membranes, the wing was snap-frozen in liquid nitrogen and then broken perpendicular to the main veins of the wing using tweezers. Broken wings were attached to metallic discs at one end, to enable the exposed end to face away from the substratum, enabling optimum imaging. Samples were then sputter coated with gold in an identical manner as used above.

### Synchrotron Radiation Fourier-transform Infrared Spectrometry (SR-FTIR)

The distribution of organic functional groups present across the wing membrane before and after chloroform extraction was analyzed using SR-FTIR, on the Infrared microscopy beamline at the Australian Synchrotron. The samples were scanned in transmission mode over several areas of approximately 50 µm × 50 µm using a Bruker Hyperion 2000 FTIR microscope (Bruker Optic GmbH, Ettlingen, Germany), equipped with a narrow-band, high-sensitivity mercury cadmium telluride detector. A long-pass filter selected the detection range from 4950 cm^−1^ to 750 cm^−1^ and the microscope was fitted with a sample chamber purged with dry air to maintain low constant humidity (approx 20%). OPUS version 6.5 software was employed for operating the microscope and spectrometer as well as for analyzing the data.

### X-ray Photoelectron Spectroscopy Depth Profiling

A VG ESCALAB 220i-XL X-ray Photoelectron Spectrometer equipped with a hemispherical analyzer was used for XPS data acquisition. Samples underwent alternating data acquisition and ion beam etching cycles. Monochromatic Al Kα X-rays (1486.6 eV) at 220 W (22 mA and 10 kV) were used as incident radiation for data acquisition. Survey scans were carried out at pass energies of 100 eV over a binding energy range of 1200 eV. Base pressure in the analysis chamber was below 7.0 × 10^−9^ mbar, and during sample depth profile analysis rose to 1.5 × 10^−7^ mbar. Ion beam etching was performed using a 4 keV Argon beam over a 2 mm × 2 mm area for intervals of 120 seconds. Data acquisition and etching was performed for 5 cycles, with a total etching time of 3000 seconds. XPS data were fitted using mixed Gaussian Lorentzian peak shape and linear background subtraction with CasaXPS.

### Gas Chromatography-mass Spectrometry (GC-MS)

Prior to GC-MS analysis, any hydroxyl-containing components of the wing extracts (e.g. alcohols and carboxylic acids) were converted to trimethylsilyl (TMSi) derivatives by means of reaction with N,N-bis-trimethylsilyl-triflouro-acetamide (BSTFA, C_8_H_21_NOSi_2_) in the presence of pyridine for 45 minutes at 70°C according to previously optimized laboratory protocols [8], [9], [21], [31]–[33]. The composition of the sample extract was determined by injecting 1 µL of the solution onto a capillary GC column (Rxi-5SIL MS column fused silica; column 30.0 m, 0.25 mm i.d., df = 0.25 µm, Restek Corporation, Bellefonte PA, U.S.A.), using He as the carrier gas at constant flow of 1.4 mL min^−1^ and a mass spectrometric detector (GCMS-QP2010, Shimadzu Scientific Instruments, Columbia, U.S.A). The chromatograph was programmed as follows: on-column injection at 50°C, oven at 50°C for 5 minutes, then increasing to 280°C at the rate 3°C min^−1^, and then held for 30 minutes at 280°C. Mass spectra were recorded from m/z 14 to 800. The percentage composition reported for each component is based on the peak area from the Total Ion Current (TIC) chromatogram without standardization. A standard mixture of *n*-alkanes (C_8_–C_40_) in chloroform was used as an external standard to verify the retention times [34]. The retention indices (RI) for each component were calculated based on:
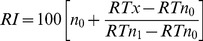
(1)where n_0_ is the number of carbons for an *n*-alkane standard and n_1_ corresponds to the next *n*-alkane in the series. RT_x_, RT_n0_ and RT_n1_ are the retention times for the component being measured relative to the two closest *n*-alkane standards. The RI was compared with retention index databases [35], [36] to identity the components not contained in the Wiley 7^th^ Edition mass spectral database.

## Results and Discussion

**Figure 1:**
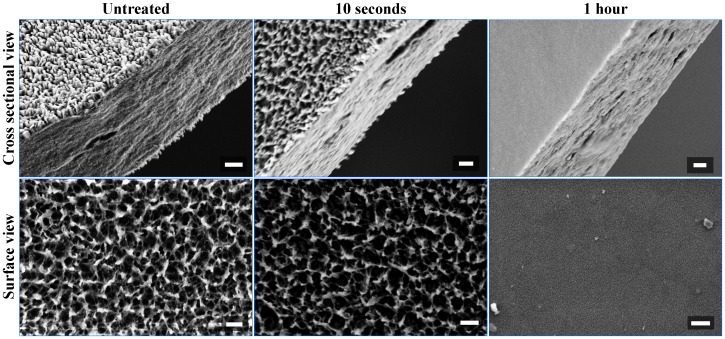
Scanning electron micrographs of *Hemianax papuensis* wing membranes. Cross-sectional (a, c, e) and surface view (b, d, f) images were taken of each wing before chloroform extraction (a, b), after 10 s extraction (c, d), and 1 hr extraction (e, f). Loss of surface structure is visible on the wings subjected to chloroform extraction; no structure is evident after extraction for 1 hr, whereas the internal wing structure appears unchanged. Scale bars = 400 nm.

The insect wing cuticle is composed of two main layers: the epicuticle and the procuticle, both of which can be further divided into two distinct sub-layers [14], [26], [37], [38]. In the case of the epicuticle, these two sub-layers are referred to as outer epicuticle and inner epicuticle. Chloroform extraction times of 10 seconds and one hour were chosen to extract lipids from the outer epicuticle and the entire epicuticle, respectively. Scanning electron micrographs confirmed that an extraction period of 10 seconds was appropriate to extract the outer epicuticle of the wing. It can be seen from surface view of untreated and 10 seconds extracted sample (Figure 1) that the wing surface structure remained largely unchanged after the 10 s extraction period. The nanostructures on the wing surface are still clearly visible; however there is increased space between individual nanostructures after 10 seconds of exposure to chloroform. In contrast, the wing surface after one hour of chloroform extraction appears completely devoid of nanostructures. This observation alone demonstrated that the chemical components responsible for forming the nanostructures on the dragonfly wings are highly chloroform-soluble. Cross sectional views of the wing were also obtained in order to obtain insight into the internal structure of the dragonfly wing membrane. Surface view and cross sectional view are presented for comparison and observation of any changes on the wing surface structure caused by liquid nitrogen. Liquid nitrogen is an efficient method for fast freezing of biological samples. This method has been applied previously on dragonfly wings to obtain fresh and planar fracture cross-sectional surfaces of the membranes in the direction of the thickness by Song *et al*. [39]. No obvious differences were observed in the structure of the bulk of the wing membrane as a result of the chloroform extraction. Also, as seen in cross-sectional views, the wing membrane is constructed in three main layers, i.e., epicuticular layers at dorsal and ventral surfaces, and intracuticular layer where the bulk is located.

**Figure 2:**
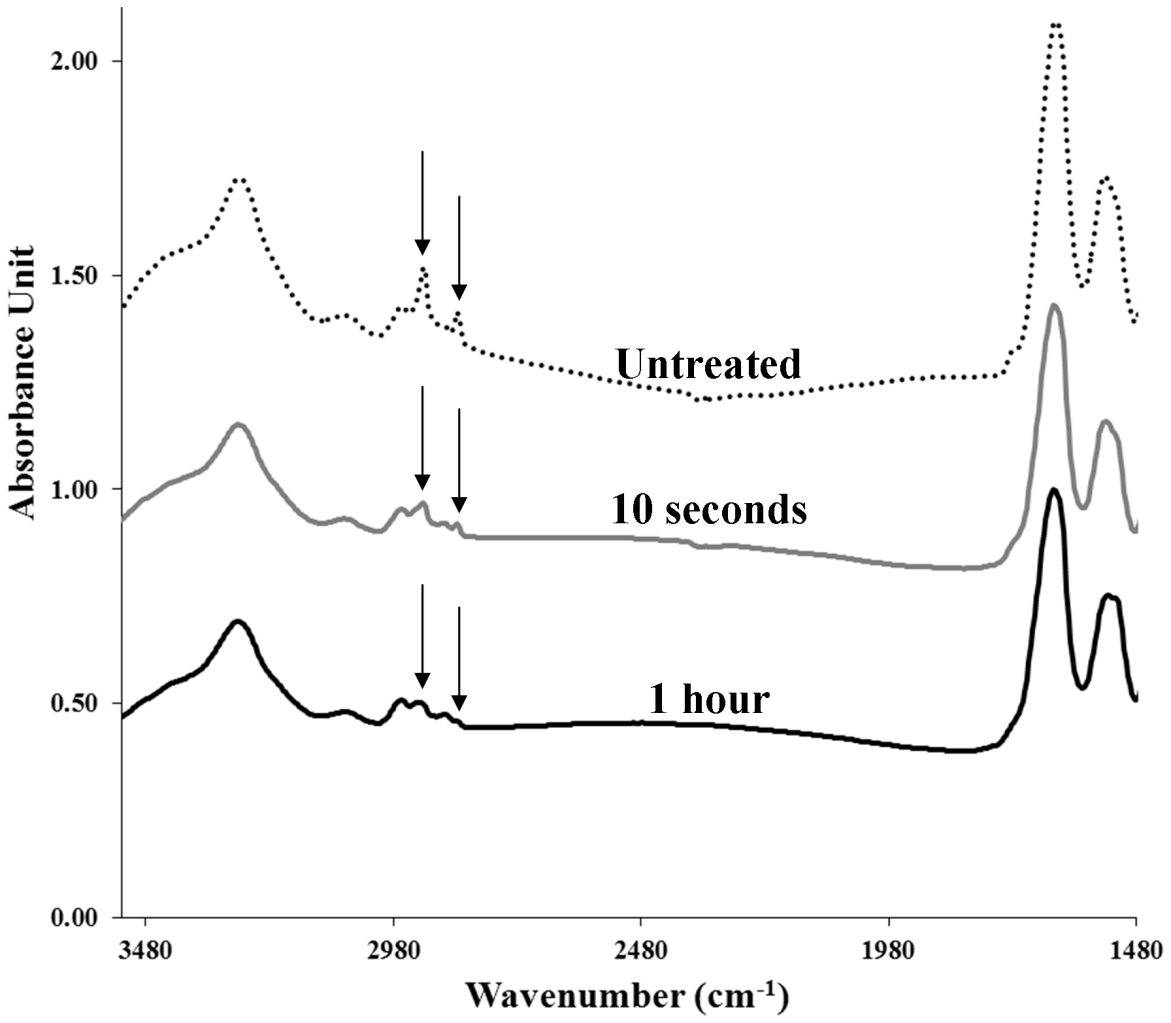
Representative infrared spectra of untreated and chloroform extracted wing membranes of *Hermianax papuensis*. The intensity of the CH (as indicated) stretching bands decrease successively with extended extraction time. Spectra were acquired in transmission mode.

Synchrotron-radiation FTIR was employed to obtained high resolution spectra of the total chemical composition across the wing membrane. Synchrotron radiation allows for the generation of small infrared beam spot-sizes which enable analysis of specific areas of the wing. Multiple scans can be conducted in short period of time within a certain area to validate the consistency of the chemical composition as well as the reliability of quantitative changes of the chemistry over different chloroform extraction times. The characteristic FTIR spectra of the chloroform-extracted wings contained three major groups of bands (Figure 2, Table 1). The two most intense bands, located at approximately 1630 cm^−1^ and 1530 cm^−1^ correspond to a carbonyl stretching vibration and an NH in-plane bending vibration, respectively. The presence of both of these bands is most likely explained by the peptide linkages in the structural protein components in the bulk of the wing membrane [40]–[43]. A significant OH stretching peak is also observable between 3200–3500 cm^−1^, which includes a contribution from an NH stretching band. These bands are also likely to be due to the bulk structural components of the wing, i.e. proteins and chitin. The third group of bands is located between 2800 cm^−1^ and 3000 cm^−1^, and is associated with CH stretching vibrations. Of particular interest are the two peaks at 2830 cm^−1^ and 2930 cm^−1^, which correspond to symmetric and antisymmetric CH_2_ stretching vibrations. These two bands are of relatively high intensity in the spectra recorded for the untreated wings, which is indicative of long chain aliphatic hydrocarbons, i.e. lipid components. After 10 seconds of chloroform extraction, the intensity of both peaks drop significantly, indicating that the lipids were successfully extracted by the chloroform. After one hour of chloroform extraction, the peaks drop further, but are still present. This is to be expected, as proteins, chitin and other organic molecules that constitute part of the wing membrane will contribute to absorption in this region of the spectrum [11], [15]–[19], [22], [25], [41], [44]–[47].

**Table 1:** Frequencies and assignments of the major absorption peaks found in the IR spectra of *Hermianax papuensis.*

Band position (cm^−1^)	Functional groups	Characteristic
1480–1580	Amide II	• Out-of-phase combination of the NH in plane bend and the CN stretching vibration• Carbonyl stretching band due to ketone, carboxylic acid [42]
1570–1700	Amide I Carbonyl groups	• C = O stretch vibration causes CCN deformation and the NH in-plane bend
1700–1750	Ester	• Carbonyl stretching band
2800–3000	Alkanes or alkyl groups	• CH_2_, CH_3_ symmetric and asymmetric stretch [78]
3000–3100	Unsaturated and aromatic C-H	• CH stretch (C is sp^2^) [79]
3200–3500	O-H stretch from free alcohol or phenol	• O–H stretching, which can be due to carbohydrates, carboxylic acids, amides or alcohols/phenols [79]

**Figure 3:**
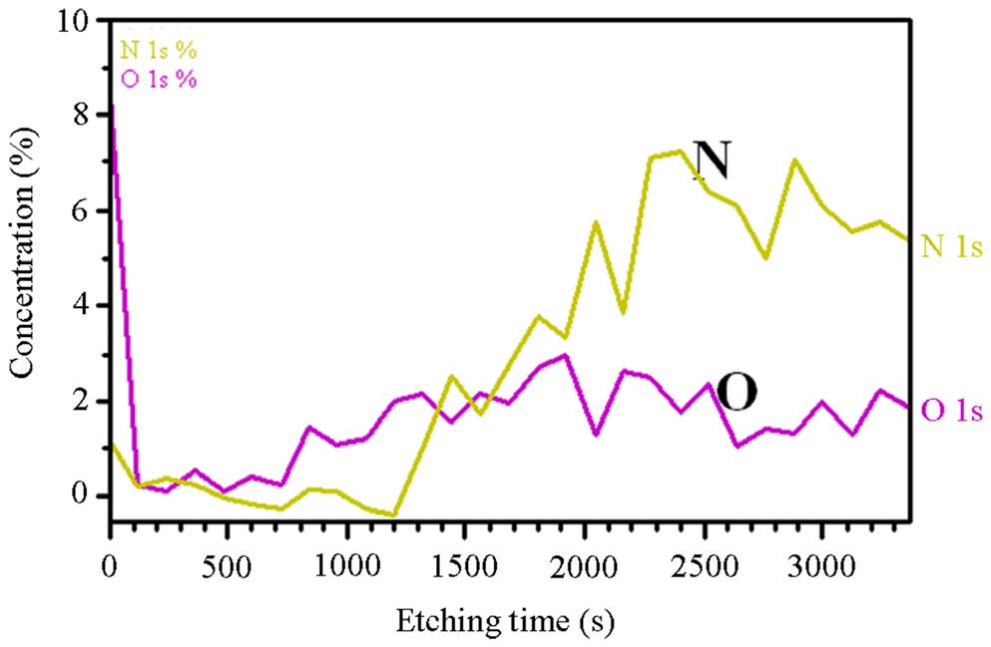
Atomic proportions of oxygen and nitrogen in the wings of *Hemianax papuensis*.

Unfortunately, the spatial resolution that can be obtained using Fourier-transform infrared spectroscopy is too low to enable a detailed chemical analysis of the nanoscale surface features. Conversely, x-ray photoelectron spectroscopy (XPS) is capable of nanometer resolution in the z-dimension [48]–[51]. Depth-profiling XPS spectra obtained by alternating data acquisition and ion-beam etching were found to be dominated by carbon. Some interesting observations could be made based on the oxygen and nitrogen profiles (Figure 3). After the first etching cycle, the oxygen content on the wing surface decreased to almost zero. After 6 etching cycles, the detected oxygen levels began to increase and quickly stabilized at around 2%. The contribution of nitrogen components was insignificant until after the tenth etching cycle, after which it increased to around 6%. This is an indication that after 10 etching cycles the protein and chitin structural components in the bulk of the wing was in close enough proximity to the surface to be detected in the spectra. Based on this observation, a tentative estimate of the etching rate can be made; dragonfly wings are typically ∼3 µm thick, [11], [12], [39], [52] and from the images presented in Figure 1 the epicuticular layers of the wings of *H. papuensis* can be roughly estimated to account for one tenth of the cross-section of the wing. Thus in 10 etching cycles, i.e. 20 minutes, the wings were etched to a depth of approximately 300 nm, which corresponds to an etching rate of 15 nm min^−1^.

**Figure 4:**
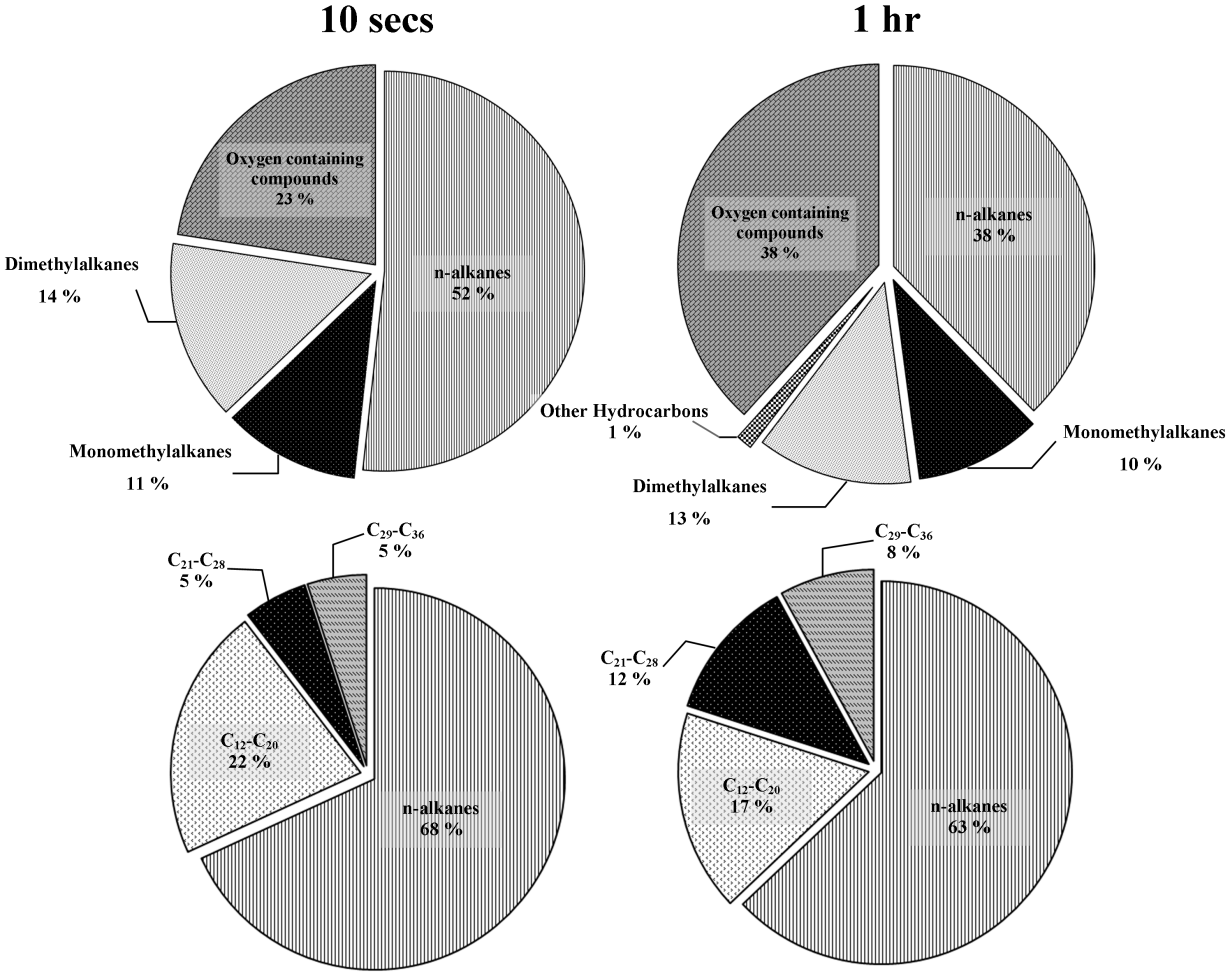
Relative proportions of the major compound classes and chain length of dragonfly wing epicuticle components.

While surface analytical techniques are very useful for generating maps and locating specific chemical functionalities, they usually return a signal or spectrum that is the sum of all of the chemical components at that interface, which can make detailed molecular identification difficult. To overcome this problem, a chromatographic analysis of the lipid extracts was conducted using GC-MS. Analysis of the GC-MS data revealed that the major lipid components of the wings were comprised mostly of aliphatic hydrocarbons, with a significant contribution from oxygenated compounds (Figure 4, Table S1). Straight-chain alkanes made up approximately 50% of the compounds detected in the 10 second chloroform extracts, with an additional 25% being monomethylated (11%) and dimethylated alkanes (14%). The remaining 23% of the extract could be attributed to oxygenated compounds; more specifically this group was almost entirely comprised of hexadecanoic acid. Hexadecanoic acid, or palmitic acid, is a fatty acid widely distributed among plants, animals and insects [31], [53]–[59]. After 1 hour of chloroform extraction, the proportion of oxygenated compounds increased to 38% of the identified compounds, largely due to the detection of octadecanoic acid. Octadecanoic acid was entirely undetected in the 10 second extracts, therefore it is likely that octadecanoic acid was primarily situated deeper within the wing membrane, beneath the epicuticular surface.

**Figure 5:**
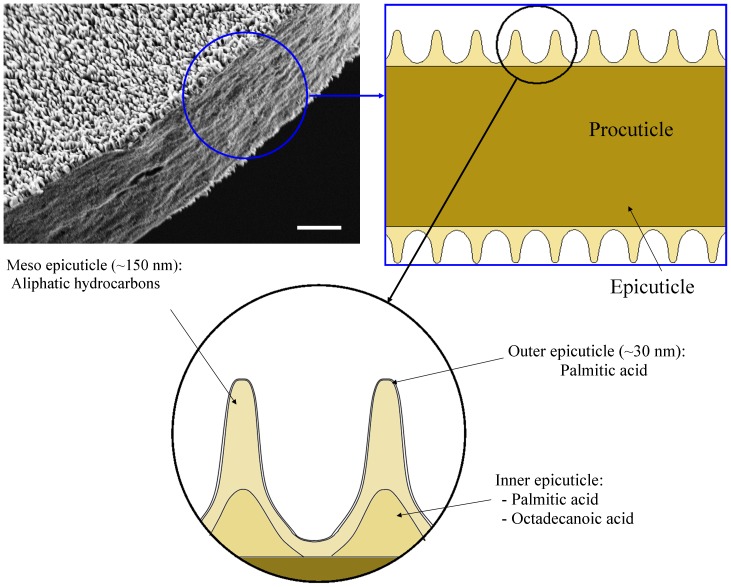
Proposed model of the epicuticle of *Hemianax papuensis* wing membranes. Three layers are contained within the epicuticle: the outer epicuticle, the meso epicuticle and the inner epicuticle.

When one considers the XPS depth profiling data in parallel with the GC-MS data, a tentative model of the molecular organization of the wing membrane can be proposed. According to the XPS analysis, the oxygen content dropped to 0 after just one etching cycle (Figure 3). The GC-MS analysis suggested that the only significant oxygen containing compound in the 10 second lipid extracts was hexadecanoic acid, which suggested that there was a thin layer at the outer surface of the wing that contained hexadecanoic acid. One hour lipid extracts contained a significant proportion of octadecanoic acid, along with an increased proportion of hexadecanoic acid. XPS data indicated that the oxygen content only began to increase again after 6 etching cycles, suggesting the presence of a deeper layer that contained both hexadecanoic acid and octadecanoic acid. Aliphatic hydrocarbons were detected throughout the wing membrane and in the regions between these two fatty acid-containing layers, they were the only compounds detected. In addition, based on the etching rate given above, the layer thicknesses can be estimated. Exposure of biological samples to ultra-high vacuum (UHV) can cause dehydration which may lead to the collapse of the intracuticle. However, this study focuses on the epicuticular layer of the wing which is much less hydrated since they do not contain sugar or protein [60]. Based on this information, a model of the epicuticle of *H. papuensis* is proposed that includes three distinct layers, which has never previously been reported for insect epicuticles (Figure 5). It is proposed that the new layer be referred to as the meso epicuticle, and that it is positioned between the outer and inner epicuticles.

The aliphatic hydrocarbon constituents of both the 10 second and the 1 hour extracts contained several main compounds in relatively high abundance. In addition, both extracts contained significant portions of *n*-alkanes with even numbers of carbon atoms ranging from C_14_ to C_30_. Biosynthesis of *n*-alkanes occurs by the addition of acetyl groups to a fatty acyl precursor conjugated to co-enzyme A [59], [61]. The chain-lengths of the resulting hydrocarbons are therefore in multiples of 2 and are dependent on the length of the fatty-acyl chain of the precursor. The composition of the minor constituents varied significantly between the two extracts, with many more compounds being detected in small quantities after one hour of extraction. Most of these minor constituents were identified as various methyl-substituted alkanes, however three alkenes were detected in the 1 hour extract; 1-hexadecene, 1-octadecene, and 1-eicosene. In addition, two esters, one aldehyde and one terpenoid were also detected in very small proportions (see Table S1 for details).

All of the components identified through GCMS analysis were found to possess low surface energy, which is one of the factors contributing to the superhydrophobicity of the wing surface, as is the hierarchical surface structure, which is the result of self-assembly of the different hydrophobic compounds onto the wing membrane. During the pupal stage of growth of the dragonfly, these compounds are secreted by epithermal cells and transported to the surface through a system of pore canals [14], [62]. Our hypothesis for the formation of the regular pillar-liked structure onto the wing surface is that during the metamorphosis stage, these compounds reorganise themselves and self-assemble into a particular nanostructure. Previous studies that have been performed on plant surfaces have revealed that substances possessing different chemical compositions will result in the formation of different surface morphologies, which supports the hypothesis of self-organisation [7], [20].

Plant cuticles have similar composition to that of the dragonfly wing epicuticle reported here. The works of Bharat Bhushan [63]–[68], Kerstin Koch [7], [69]–[71], and Hans J. Ensikat [72] in particular have contributed greatly to the knowledge of the composition of plant cuticular waxes. Plant waxes also contain various derivatives of long aliphatic hydrocarbons, including long chain alkanes and fatty acids [20], [71]–[76]. Koch *et al.* showed that the cuticular wax of lotus leaves also contributes in forming the surface nanostructures [69]. It is well established that there is high variation in the proportions of many of the chemical components in plant waxes between plant species and even between different organs of the same plant [77].

### Conclusions

Dragonfly wings are covered with nanoscale surface structures that afford the wings self-cleaning and superhydrophobic properties. While the outer epicuticle is generally accepted to be composed of lipids and waxes, the compounds that form the surface nanostructures have not previously been identified. Here, we characterized the epicuticular lipids of dragonfly wings using a combination of three complementary analytical techniques: Synchrotron-sourced Fourier-transform infrared microspectroscopy (FTIR), gas chromatography-mass spectrometry (GCMS) and x-ray photoelectron spectroscopy (XPS). FTIR spectra collected for wings after the surface lipids had been extracted with chloroform were found to contain characteristic absorption bands for amide, ester and aliphatic hydrocarbons. Importantly, the CH_2_ stretching bands, which were indicative of epicuticular lipids, were found to decrease in intensity with increasing extraction time. XPS depth profiling, in conjunction with GCMS analysis of the chloroform extracts, enabled the detection of three distinct layers within the epicuticle: the outer epicuticle, which contained hexadecanoic acid, the meso epicuticle, which was composed of aliphatic hydrocarbons, and the inner epicuticle, which contained hexadecanoic acid and octadecanoic acid. The identification of a third sub-layer of the epicuticle of insect wings has never been previously reported. The data presented here indicate that the nanostructures on the surface of dragonfly wings are composed primarily of aliphatic hydrocarbons, with an outer layer composed largely of fatty acids. This information will be extremely valuable in future attempts to replicate dragonfly wing structures and properties onto model substrates for industrial applications.

## Supporting Information

Table S1 **Individual wax components of the** ***Hemianax papuensis*** **wing epicuticle isolated and identified by GCMS.** Components that could not be unambiguously identified are grouped together.
(DOCX)
