# Supplementary material for: Molecular Organization of the Nanoscale Surface Structures of the Dragonfly Hemianax papuensis Wing Epicuticle
Source: PLoS One. 2013 Jul 9;8(7):e67893. doi: 10.1371/journal.pone.0067893 (PMC3706462; doi:10.1371/journal.pone.0067893)
Supplement: Table S1 — Individual wax components of the Hemianax papuensis wing epicuticle isolated and identified by GCMS. Components that could not be unambiguously identified are grouped together. (DOCX) [file pone.0067893.s001.docx]

**Supporting Information**.

Molecular organization of the nanoscale surface structures of the dragonfly Hemianax papuensis wing epicuticle

Elena P. Ivanova, Song Ha Nguyen, Hayden K. Webb, Jafar Hasan, Vi Khanh Truong, Robert N. Lamb, Xiaofei Duan, Mark J. Tobin, Peter J. Mahon, Russell J. Crawford

**Table S1.** Individual wax components of the *Hemianax papuensis* wing epicuticle isolated and identified by GCMS. Components that could not be unambiguously identified are grouped together.

| Formula | RI | Chemical name | 10 s | 1 h |
| --- | --- | --- | --- | --- |
| C_7_H_12_O | 965 | 2-Heptenal | 0.15 | 0.17 |
| C_12_H_26_ | 1200 | n-dodecane |  | 0.21 |
| C_14_H_30_ | 1371 | 3-methyltridecane |  | 0.05 |
| C_14_H_30_ | 1400 | n-tetradecane | 5.45 | 2.33 |
| C_15_H_32_ | 1540 | 3-methyltetradecane |  | 0.12 |
| C_16_H_34_ | 1565 | 2-methylpentadecane |  | 0.014 |
| C_16_H_34_ | 1571 | 3-methylpentadecane | 1.47 | 0.69 |
| C_16_H_32_ | 1593 | 1-hexadecene |  | 0.11 |
| C_16_H_34_ | 1600 | n-hexadecane | 10.57 | 5.66 |
| C_18_H_38_ | 1728 | 8-ethylhexadecane |  | 0.085 |
| C_18_H_38_ | 1728 | 7-ethylhexadecane |  | 0.085 |
| C_18_H_38_ | 1733 | 6-ethylhexadecane |  | 0.04 |
| C_18_H_38_ | 1744 | 5-methylheptadecane |  | 0.022 |
| C_18_H_38_ | 1744 | 7-methylheptadecane |  | 0.022 |
| C_18_H_38_ | 1765 | 2-methylheptadecane |  | 0.03 |
| C_18_H_38_ | 1772 | 3-methylheptadecane | 4.2 | 1.51 |
| C_18_H_36_ | 1793 | 1-octadecene |  | 0.07 |
| C_18_H_38_ | 1800 | n-octadecane | 10.28 | 6.69 |
| C_18_H_38_ | 1799 | 6,12-dimethylheptadecane | 10.28 | 6.69 |
| C_17_H_34_O_2_ | 1853 | Pentadecyl acetate |  | 0.024 |
| C_18_H_38_ | 1888 | 7,12-dimethylhexadecane |  | 0.12 |
| C_20_H_42_ | 1888 | 7,12-dimethyloctadecane |  | 0.12 |
| C_20_H_42_ | 1895 | 6,13-dimethyloctadecane |  | 0.04 |
| C_20_H_42_ | 1915 | 6,7-dimethyloctadecane |  | 0.095 |
| C_20_H_42_ | 1921 | 5,6-dimethyloctadecane |  | 0.06 |
| C_20_H_42_ | 1928-1933 | 6,10,14-trimethyloctadecane |  | 0.17 |
| C_20_H_42_ | 1944 | 7-methylnonadecane |  | 0.085 |
| C_20_H_42_ | 1944 | 8-methylnonadecane |  | 0.085 |
| C_20_H_42_ | 1966 | 2-methylnonadecane |  | 0.22 |
| C_20_H_42_ | 1973 | 3-methylnonadecane | 2.12 | 1.7 |
| C_20_H_40_ | 1994 | 1-eicosene |  | 0.032 |
| C_20_H_42_ | 2000 | n-eicosane | 8.5 | 6.3 |
| C_18_H_34_O | 2004 | 1-octadecenal |  | 0.014 |
| **C_16_H_32_O_1_** | **2046** | **Hexadecanoic acid** | **24.88** | **32.73** |
| C_22_H_46_ | 2116 | 7-ethyleicosane |  | 0.22 |
| C_22_H_46_ | 2133 | 11-methylheneicosane |  | 0.08 |
| C_22_H_46_ | 2142 | 5-methylheneicosane |  | 0.06 |
| C_22_H_46_ | 2142 | 7-methylheneicosane |  | 0.06 |
| C_22_H_46_ | 2142 | 9-methylheneicosane |  | 0.06 |
| C_10_H_16_O_2_ | 2162 | (E)-3,7-dimethyl-2,6-octadienoic acid |  | 0.07 |
| C_22_H_46_ | 2170 | 3-methylheneicosane | 1.72 | 1.86 |
| C_23_H_48_ | 2170 | 7,11-dimethylheneicosane | 1.72 | 1.86 |
| C_22_H_46_ | 2200 | n-docosane | 6.9 | 6.68 |
| C_18_H_36_O_2_ | 2244 | Octadecanoic acid |  | 12.96 |
| C_24_H_50_ | 2282 | 9-propylheneicosane |  | 0.174 |
| C_24_H_50_ | 2315 | 9-ethyldocosane |  | 0.27 |
| C_24_H_50_ | 2324 | 2-methyltricosane |  | 0.02 |
| C_24_H_50_ | 2373 | 3-methyltricosane | 1.57 | 2.04 |
| C_25_H_52_ | 2415 | 12-ethyltricosane |  | 0.07 |
| C_25_H_52_ | 2431 | 11-methyltetracosane | 0.19 | 0.17 |
| C_26_H_54_ | 2480 | 12-propyltricosane |  | 0.11 |
| C_26_H_54_ | 2507 | 11-ethyltetracosane |  | 0.12 |
| C_27_H_56_ | 2525 | 11,15-dimethylpentacosane |  | 0.05 |
| C_26_H_54_ | 2532 | 9-methylpentacosane |  | 0.12 |
| C_26_H_54_ | 2532 | 10-methylpentacosane |  | 0.12 |
| C_26_H_54_ | 2532 | 11-methylpentacosane |  | 0.12 |
| C_26_H_54_ | 2532 | 13-methylpentacosane |  | 0.12 |
| C_26_H_54_ | 2544 | 7-methylpentacosane |  | 0.07 |
| C_26_H_54_ | 2544 | 5-methylpentacosane |  | 0.07 |
| C_27_H_56_ | 2544 | 9,13-dimethylpentacosane |  | 0.07 |
| C_27_H_56_ | 2570 | 7,11-dimethylpentacosane |  | 0.01 |
| C_26_H_54_ | 2575 | 3-methylpentacosane | 0.72 | 1.57 |
| C_27_H_56_O | 2595 | Heptacosanol |  | 0.04 |
| C_26_H_54_ | 2600 | n-hexacosane | 9.95 | 7.1 |
| C_28_H_58_ | 2704 | 2,6-dimethylhexacosane |  | 0.08 |
| C_29_H_60_ | 2767 | 9,11-dimethylheptacosane |  | 0.043 |
| C_29_H_60_ | 2767 | 11,15-dimethylheptacosane |  | 0.043 |
| C_29_H_60_ | 2767 | 7,11-dimethylheptacosane |  | 0.043 |
| C_28_H_58_ | 2800 | n-octacosane | 2.95 | 4.94 |
| C_29_H_60_ | 2807 | 3,15-dimethylheptacosane |  | 0.012 |
| C_29_H_60_ | 2807 | 3,11-dimethylheptacosane |  | 0.012 |
| C_29_H_60_ | 2807 | 3,9-dimethylheptacosane |  | 0.012 |
| C_29_H_60_ | 2900 | n-nonacosane |  | 0.078 |
| C_29_H_60_ | 2973 | 3-methyloctacosane | 0.36 | 1.03 |
| C_30_H_62_ | 3000 | n-triacontane | 2.42 | 3.11 |
| C_32_H_66_ | 3097 | 2,10-dimethyltriacontane | 0.5 | 0.08 |
| C_32_H_66_ | 3097 | 2,12-dimethyltriacontane | 0.5 | 0.08 |
| C_31_H_64_ | 3100 | n-hentriacontane | 0.09 | 0.053 |
| C_33_H_68_ | 3172 | 7,23-dimethylhentriacontane | 0.07 | 0.076 |
| C_32_H_66_ | 3172 | 3-methylhentriacontane | 0.07 | 0.076 |
| C_33_H_68_ | 3172 | 7,25-dimethylhentriacontane | 0.07 | 0.076 |
| C_32_H_66_ | 3200 | n-dotriacontane | 0.34 | 2.14 |
| C_35_H_72_ | 3369 | 7,19-dimethyltritriacontane | 0.135 | 0.035 |
| C_35_H_72_ | 3369 | 7,21-dimethyltritriacontane | 0.135 | 0.035 |
| C_34_H_70_ | 3372 | 3-methyltritriacontane |  | 0.035 |
| C_35_H_72_ | 3372 | 7,23-dimethyltritriacontane |  | 0.035 |
| C_34_H_70_ | 3400 | n-tetratriacontane | 0.11 | 0.092 |
| C_32_H_52_O_2_ | 3578 | Retinyl dodecanoate | 0.07 | 0.03 |
| C_36_H_74_ | 3600 | n-hexatriacontane |  | 0.035 |

%Composition based on measured TIC peak areas without standardization.
